# Psychometric evaluation and community norms of the PHQ-9, based on a representative German sample

**DOI:** 10.3389/fpsyt.2024.1483782

**Published:** 2024-12-12

**Authors:** Sören Kliem, Cedric Sachser, Anna Lohmann, Dirk Baier, Elmar Brähler, Harald Gündel, Jörg M. Fegert

**Affiliations:** ^1^ Department of Social Welfare, Ernst-Abbe-Hochschule Jena - University of Applied Sciences, Jena, Germany; ^2^ Department for Child and Adolescent Psychiatry/Psychotherapy, University Clinic for Psychosomatic Medicine and Psychotherapy Ulm, Ulm, Germany; ^3^ Institute of Delinquency and Crime Prevention, Zurich University of Applied Sciences, Zurich, Switzerland; ^4^ Department of Psychosomatic Medicine and Psychotherapy, University Medical Center of the Johannes Gutenberg University Mainz, Mainz, Germany; ^5^ Department of Psychosomatic Medicine and Psychotherapy, Ulm University Medical Center, Ulm, Germany

**Keywords:** PHQ-9, major depression, self-report questionnaire, population norms, psychometrics, measurement invariance

## Abstract

**Background:**

The Patient Health Questionnaire (PHQ-9) is a popular tool for assessing depressive symptoms in both general and clinical populations. The present study used a large representative sample of the German adult population to confirm desired psychometric functioning and to provide updated population norms.

**Methods:**

The following psychometric properties were assessed: (i) Item characteristics (item means, standard deviations and inter-item correlations), (ii) Construct validity (correlations of the PHQ-9 sum-score with scores obtained from instruments assessing depression, anxiety and somatization (GAD-7, BSI-18), (iii) Internal consistency (coefficient omega), (iv) Factorial validity (via confirmatory factor analysis of the assumed one factorial model) as well as (v) Measurement invariance (via multi-group confirmatory factor analyses across gender, age, income and education).

**Results:**

The study found that the PHQ-9 had sound psychometric properties in terms of internal consistency and construct validity, and that measurements obtained with the tool could be compared across gender and age.

**Limitations:**

Despite using a representative sample, the response rate was only 42.6%. Furthermore, diagnostic efficiency cannot be assessed as there were no clinical interviews conducted. Conclusion: The updated population based norms, which are presented for the total sample as well as separated by gender and various age-groups, provide a useful reference for clinical practice and epidemiological research.

## 1 Introduction

Major depression is a common mood disorder that requires brief and comprehensive screening instruments for its detection and assessment in both clinical and research settings (1). The 9-question Patient Health Questionnaire (PHQ-9) scale is such a tool which corresponds to DSM-IV major depressive criteria and was developed as a self-administered questionnaire for use in primary care setting (2). It is widely established for detecting the presence and severity of depression. Various cut-off scores have been suggested that optimize sensitivity, specificity as well as positive and negative predictive values obtained via diagnostic clinical interviews (2–4). A recent individual participant data meta-analysis (5) extending the work of (3), encompassing 100 studies and 44,503 participants, evaluated the accuracy of the PHQ-9 against various diagnostic methods, including semistructured and fully structured interviews, as well as the Mini International Neuropsychiatric Interview (MINI). The analysis revealed that the commonly used cut-off score of ≥10 optimized both sensitivity (0.85, 95% CI 0.79-0.89) and specificity (0.85, 95% CI 0.82-0.87) when compared with semistructured diagnostic interviews.

Studies like these demonstrate that the PHQ-9 is a brief and effective tool for depression screening While it may have limitations compared to more detailed measures like the CIDI (6) or BDI-II (7), particularly in specialized clinical settings. Nevertheless, its ease of use and its alignment with diagnostic criteria of major depressive disorder makes it ideal for routine screening in general practice.

The fact that it is well validated and freely available in many languages also makes it a popular tool in epidemiological studies of mental health and psychological distress [e.g., (1, 8–10)]. It has proven useful across socioeconomic backgrounds (11) and cultures. More recently, the PHQ-9 has also been used extensively assessing the mental health burden related to the COVID-19 pandemic [e.g., (12–15)].

Normative values of a questionnaire are crucial for assessing the level of distress in individuals and groups of patients. While the PHQ-9 has been widely used in various populations and settings, the research literature on normative scores obtained from representative general population samples is very limited [e.g., (16–18)]. The only large normative study conducted with adults from the general population in Germany is based on data from 2003-2008 (19).

As the PHQ-9 uses a sum-score for the interpretation of symptom severity, it is paramount to confirm the factor structure to ensure that this form of aggregation is appropriate (20). The PHQ-9 factor structure has been frequently debated in the literature, with the majority of studies suggesting a one factorial structure and the occasional mention of two or more highly correlated factors (21).

With depression levels varying across several demographic groups, it is furthermore important to confirm measurement invariance in order to access whether findings are comparable across various sub-populations (11, 22). Especially gender differences have been identified as substantial by meta-analyses (23).

Therefore, the aim of this study was to assess the psychometric properties of the PHQ-9 in a large representative sample of the general population and provide updated German population norms.

Moreover, due to the widespread use of the PHQ-9, it is important to capture any potential shifts in item behavior in the general population. The current data obtained from a large representative community sample provides a valuable reference distribution for more meaningful interpretations of data obtained from other populations and settings.

## 2 Methods

### 2.1 Procedure

The PHQ-9 was presented as part of a large survey conducted by Leipzig University between December 2020 and March 2021.

The goals of the survey were (a) to assess prevalence rates of a variety of relevant physical or mental disorders and related risk behaviors (descriptive epidemiology), (b) to examine causes and conditions of these disorders (analytic epidemiology), and (c) to analyze psychometric properties and provide German population norms for clinical-psychological instruments. The survey was carried out by the contractor USUMA Markt- und Sozialforschung an independent institute for opinion and social research.

It consisted of two parts. The first part was guided by a trained interviewer and collected extensive demographic as well as household information. Survey contents in this part were based on principles of the German Statistisches Bundesamt (Federal Statistical Office).

The second part consisted of paper-based self-administered questionnaires which the participants filled in independently. Interviewers remained out of view but available for questions. Prior to their participation in the survey all participants obtained a written copy of the confidentiality agreement providing details regarding the handling of their personal data. The study followed the Declaration of Helsinki. Minimum age for participation was 16 years. All participants provided informed consent prior to the interview. For under-aged participants at least one legal guardian was informed about the sampling procedure and the survey contents. All procedures were approved by the Ethics Committee of the Medical Faculty of the University of Leipzig (Az.: 474/20-ek).

### 2.2 Sample description

**Figure 1:**
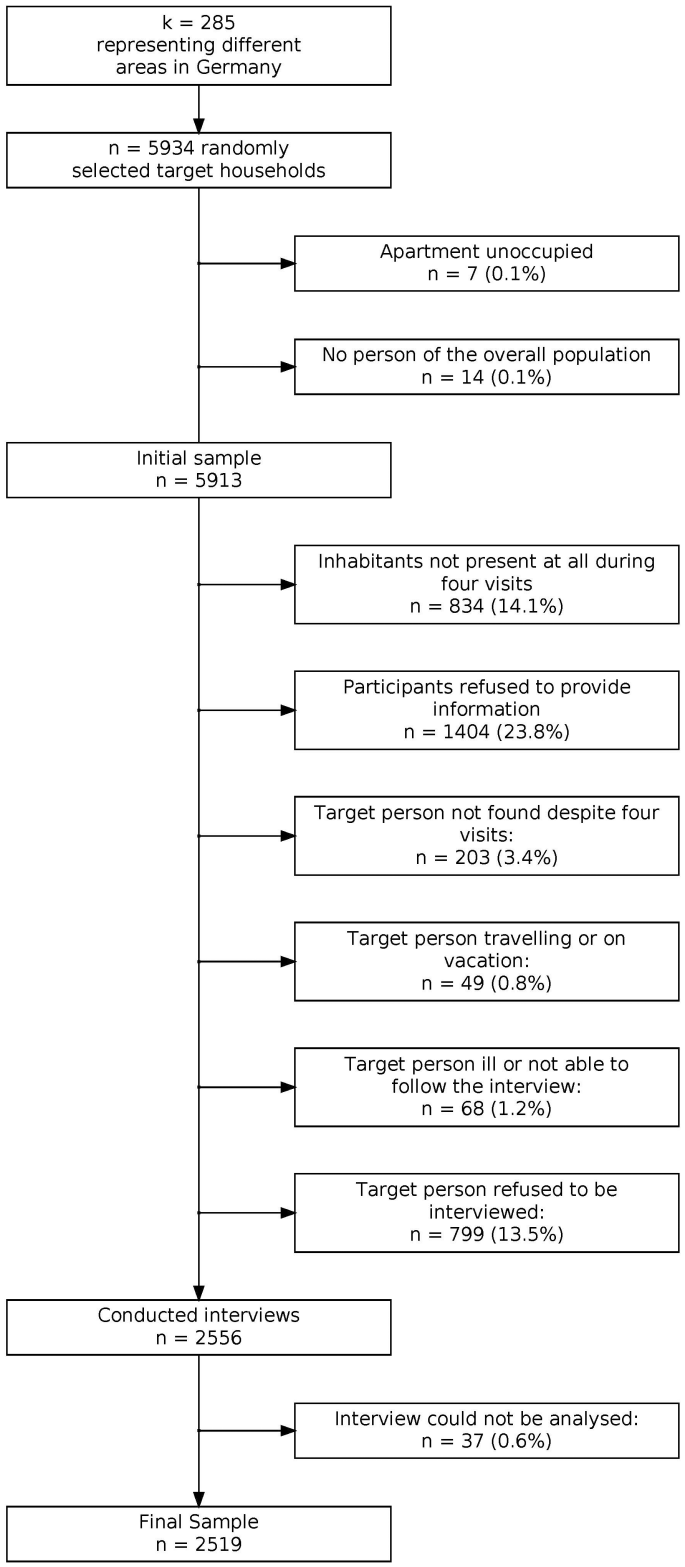
Flowchart of sampling procedure and reasons for nonparticipation.

As Germany does not keep a central population registry, representativeness of the sample was ensured by using the ADM sampling system F2F. This sampling procedure consists of three steps. In a first step, the area of the Federal Republic of Germany is divided into regions of which 258 are sampled with sampling probability proportional to the number of households. In a second step, 5676 households are selected based on a random route procedure. Finally, the target person within each household is identified using a Kish selection grid (24). Further details regarding the sampling procedure, COVID measures and sample representativeness can be found in the **Supplementary Materials** (section A). Details regarding response can be obtained from **Figure 1**. The following analyses are based on data from *N* = 2519 participants which corresponds to a response rate of 42%. **Figure 1** presents a flowchart outlining the sampling procedure and reasons for non-response. **Table 1** provides sample descriptives.

**Table 1:** Demographic characteristics of the study sample.

	Male	Female	Diverse	Total
	(N=1193)	(N=1322)	(N=4)	(N=2519)
Age (years)				
Mean (SD)	50.1 (17.7)	50.5 (18.3)	44.8 (26.5)	50.3 (18.1)
Median [Min, Max]	52.0 [16.0, 96.0]	51.0 [16.0, 96.0]	41.5 [21.0, 75.0]	51.0 [16.0, 96.0]
Age Categories				
16-24	102 (8.5%)	125 (9.5%)	2 (50.0%)	229 (9.1%)
25-34	190 (15.9%)	174 (13.2%)	0 (0%)	364 (14.5%)
35-44	174 (14.6%)	225 (17.0%)	0 (0%)	399 (15.8%)
45-54	195 (16.3%)	216 (16.3%)	0 (0%)	411 (16.3%)
55-64	244 (20.5%)	243 (18.4%)	1 (25.0%)	488 (19.4%)
65-74	190 (15.9%)	200 (15.1%)	0 (0%)	390 (15.5%)
75+	98 (8.2%)	139 (10.5%)	1 (25.0%)	238 (9.4%)
Nationality				
German	1151 (96.5%)	1271 (96.1%)	3 (75.0%)	2425 (96.3%)
not German	42 (3.5%)	48 (3.6%)	1 (25.0%)	91 (3.6%)
Missing	0 (0%)	3 (0.2%)	0 (0%)	3 (0.1%)
Marital Status				
married/living together	547 (45.9%)	527 (39.9%)	2 (50.0%)	1076 (42.7%)
married/separated	40 (3.4%)	25 (1.9%)	0 (0%)	65 (2.6%)
single	398 (33.4%)	357 (27.0%)	2 (50.0%)	757 (30.1%)
divorced	143 (12.0%)	227 (17.2%)	0 (0%)	370 (14.7%)
widowed	62 (5.2%)	181 (13.7%)	0 (0%)	243 (9.6%)
Missing Living with partner	3 (0.3%)	5 (0.4%)	0 (0%)	8 (0.3%)
living with partner	729 (61.1%)	737 (55.7%)	2 (50.0%)	1468 (58.3%)
not living with partner	444 (37.2%)	565 (42.7%)	2 (50.0%)	1011 (40.1%)
Missing Educational Attainment	20 (1.7%)	20 (1.5%)	0 (0%)	40 (1.6%)
No University Entry Qualification	921 (77.2%)	1025 (77.5%)	3 (75.0%)	1949 (77.4%)
University Entrancy Qualification	262 (22.0%)	288 (21.8%)	1 (25.0%)	551 (21.9%)
Missing Monthly per Capita Houshold Income (€)	10 (0.8%)	9 (0.7%)	0 (0%)	19 (0.8%)
Mean (SD)	2050 (997)	1900 (917)	2880 (2100)	1970 (961)
Median [Min, Max]	1750 [125, 7500]	1730 [144, 5300]	1730 [1590, 5300]	1750 [125, 7500]
Missing	22 (1.8%)	41 (3.1%)	1 (25.0%)	64 (2.5%)

### 2.3 Instruments

As the survey served multiple epidemiological purposes, only those measures that were used in the validation process are discussed in this paper. In addition to extensive demographic information (see **Table 1**), health related behavior, such as the number of sick days, doctor visits, and hospital stays, were assessed. The following measures were used for the validation of the scale at hand.

#### 2.3.1 Patient Health Questionnaire (PHQ-9)

The PHQ-9 (2) is a self-report scale, that scores depression symptoms using nine items. Participants indicate symptom frequency over the last two weeks on a four-point Likert scale from 0 (not at all) to 3 (almost every day), providing a total severity score ranging from 0 to 27. In the present study, the German version of the PHQ-9 (25) was used. The PHQ-9 showed high internal consistency in previous general population studies [*α* = 0.87 (19)].

#### 2.3.2 The General Anxiety Disorder Scale (GAD-7)

The GAD-7 (26) is a brief self-report scale with seven items assessing generalized anxiety. Each of the seven items is rated on a scale from 0 (not at all) to 3 (almost every day). The total score of the GAD-7 ranges from 0-21. The GAD-7 showed high internal consistency in previous general population studies (*α* = 0.89 (10); study at hand: study at hand: *α* = 0.90, 95% CI [0.89 - 0.91]; *ω* = 0.92, 95% CI [0.91 - 0.92]). For a psychometric evaluation of the GAD-7 based on the current data see (Kliem et al., 2024)^1^.

#### 2.3.3 The Brief Symptom Inventory (BSI-18)

The BSI-18 (27) is an 18-item short form of the Symptom-Checklist 90-R. It contains three subscales each of which comprises six items: somatization (SOMA), depression (DEPR) and anxiety (ANX). The BSI’s sum score of all 18 items can be interpreted as a Global Severity Index (GSI). The BSI-18 has shown high internal consistency in previous studies of the general population (*α* = 0.93 [GSI], 0.82 [SOMA], 0.87 [DEPR], 0.84 [ANX] (28); study at hand: *α* = 0.93, 95%CI [0.92-0.94]; *ω* = 0.94, 95% CI [0.93- 0.94]).

### 2.4 Statistical analysis

#### 2.4.1 Missing data

Proportion of missing data on the PHQ-9 items ranged from 0.30% to 0.70%. In order to address missing data, we utilized chained equation modeling as outlined in van Buuren and Groothuis-Oudshoorn (29). The imputation algorithm used the following variables: gender, age, nationality, marital status, living with a partner, educational and income as well as all items from the scales PHQ-9, GAD-7 and BSI-18 to estimate missing data. We corrected for implausible item values by employing predictive mean matching, whereby the closest observable values to the predicted values ( $\hat{y}$ ) were selected. Imputation procedures were implemented using the R package mice (29). Data analysis was carried out on one imputed data set. More details on item wise missingness can be found in the **Supplementary Material**. As a sensitivity analysis all major analyses were additionally run on the unimputed data.

#### 2.4.2 Item characteristics

We calculated mean and standard deviations for all items of the PHQ-9 in the total sample and in sub-samples of male and female participants. Cohen’s *d* was used to quantify effect sizes for group differences in item means. We also calculated inter-item correlations.

#### 2.4.3 Construct validity

To evaluate construct validity of the PHQ-9, we correlated the scale with the GAD-7 and the three BSI-18 subscales (somatization, anxiety and depression) as well as with the BSI global severity index. The following hypotheses were formulated: depression levels should be higher in individuals with (a) higher anxiety scores, and (b) higher somatization scores [e.g., Gierk et al. (30); Kliem et al. (31)].

#### 2.4.4 Internal consistency

To account for potential issues arising from unmet assumptions in the calculation of coefficient *α* (32), we assessed the internal consistency of the PHQ-9 using McDonald’s *ω*, which was computed using the semTools R package (33). This additional measure provides a more robust evaluation of internal consistency.

#### 2.4.5 Factorial validity and measurement invariance

To confirm the one-dimensional structure of the PHQ-9, we conducted confirmatory factor analyses (CFA) using the lavaan package in R statistics (34). Weighted least square means and variance adjusted estimation (WLSMV) were used, as recommended for ordered categorical response options. We also tested measurement invariance (MI) using multiple group factor analysis (MGCFA) following the procedure suggested by Wu and Estabrook (35). We used theta parameterization and identified the model by setting means and variances of latent factors to 0 and 1, respectively, item intercepts to 0, and residual variances to 1. We subsequently tested five models: (i) configural invariance (no constraints apart from those necessary for model identification), (ii) threshold invariance (constraining all thresholds to be equal), (iii) weak invariance (constraint of loadings), (iv) strong invariance (constraining of intercepts), and (v) full invariance (constraining residual variances). **Supplementary Figure C1** (in the **Supplementary Materials**) provides an overview of the structural equation models assessed. **Supplementary Table C1** (**Supplementary Materials**) provides a detailed overview of parameter constraints for each steps of the MGCFA. Chen’s (36) cut-off criteria were used, with a change of < -.01 in CFI and a change of ≥.015 in RMSEA indicating non-invariance. As Sass et al. (37) have pointed out, the cut-offs suggested by Chen are often too liberal when using WLSMV estimation. We have hence added sensitivity analyses using MLR estimation. We conducted MGCFA for the PHQ-9 across gender, age (below median age vs. above median age), age * gender, income (below median vs. above median) and educational attainment (no university entrance diploma vs. university entrance diploma). Cases classifying as neither male nor female were not included in the MGCFA for gender and age * gender due to their low number. Due to empty cells in the MGCFA of age * gender in item 9 the two highest answer categories were collapsed for this one item. The semTools package (33) for R statistics was used to conduct MI analyses.

## 3 Results

### 3.1 Item characteristics

**Supplementary Table C5** (in the **Supplementary Materials**) displays means and standard deviations for the nine items of the PHQ-9 in the total sample as well as effect sizes for mean differences regarding gender. On the item-level there was a consistent pattern of female participants exhibiting higher mean depression scores as well as higher variability on most PHQ-9 items. Effect sizes (Cohen’s *d*) of these mean differences were very small and ranged from *d* = -0.05 95% CI [-0.13,0.03] to *d* = 0 95% CI [-0.08,0.08].

### 3.2 Construct validity

To determine evidence of construct validity of the PHQ-9, correlation coefficients were calculated with related instruments. In line with our hypotheses, there were high positive correlations between the PHQ-9 and measures of somatization, anxiety and depression as assessed by BSI-18 subscales (see **Supplementary Table C7** in the Supplementary Materials). In the same vein, the GAD-7 assessing anxiety showed positive correlations with the PHQ-9.

### 3.3 Population norms

**Table 2** shows cumulative percentiles of PHQ-9 scores for the total sample. Additional norms split by gender as well as age group can be found in the Supplementary Material (see **Supplementary Tables C8**, **C9**). **Table 3** reports absolute and relative frequencies per severity category. We neither endorse nor have verified this classification but provide it as a mere descriptive to facilitate comparing results across studies.

**Table 2:** Population based norms (cumulative percentiles) of the PHQ-9 scores (total sample).

PHQ-9	Total	Age 16-24	Age 25-34	Age 35-44	Age 45-54	Age 55-64	Age 65-74	Age 75+
0	41.0	41.5	50.0	44.9	39.2	39.8	38.2	30.3
1	52.3	50.7	61.0	60.4	52.6	49.6	46.7	41.2
2	64.3	60.3	72.5	73.2	63.0	61.1	60.5	55.9
3	72.9	69.9	77.7	79.7	72.3	70.1	71.8	66.0
4	79.2	74.7	83.5	83.7	78.8	76.2	79.7	75.6
5	83.8	81.2	87.1	85.2	83.9	81.4	84.9	82.4
6	87.5	86.0	90.7	88.5	88.6	84.6	87.2	86.6
7	89.4	88.2	92.0	90.0	89.8	88.1	89.0	88.7
8	92.1	91.7	93.7	91.2	92.7	91.2	93.3	90.3
9	93.9	93.4	95.6	93.5	93.7	93.6	95.6	90.8
10	95.0	95.6	97.8	94.0	94.9	94.3	95.9	91.6
11	95.9	95.6	98.6	95.2	95.4	95.3	97.2	93.3
12	96.4	95.6	99.2	95.2	95.9	95.9	97.7	94.5
13	97.2	96.5	99.5	96.0	97.8	96.3	97.7	96.2
14	97.8	96.9	99.5	96.5	98.5	97.3	98.5	96.6
15	98.3	97.8	99.5	96.7	98.8	98.0	99.0	97.9
16	98.8	98.3	99.7	97.7	99.0	98.8	99.5	98.3
17	99.0	98.3	99.7	98.2	99.3	98.8	99.7	98.3
18	99.0	98.3	99.7	98.2	99.3	98.8	99.7	98.7
19	99.3	98.3	> 99.9	98.5	99.5	99.0	99.7	> 99.9
20	99.5	> 99.9	> 99.9	98.5	99.5	99.2	99.7	> 99.9
21	99.6	> 99.9	> 99.9	99.2	99.8	99.2	99.7	> 99.9
22	99.7	> 99.9	> 99.9	99.5	> 99.9	99.2	99.7	> 99.9
23	99.8	> 99.9	> 99.9	99.7	> 99.9	99.2	99.7	> 99.9
25	99.8	> 99.9	> 99.9	99.7	> 99.9	99.4	> 99.9	> 99.9
26	99.9	> 99.9	> 99.9	99.7	> 99.9	99.6	> 99.9	> 99.9
27	> 99.9	> 99.9	> 99.9	> 99.9	> 99.9	> 99.9	> 99.9	> 99.9

**Table 3:** PHQ-9 scores by severity category and gender.

Severity	Total		Men		Women	
	n	%	n	%	n	%
minimal (0-4)	1996	79.24	980	82.15	1014	76.70
mild (5-9)	370	14.69	152	12.74	217	16.41
moderate (10-14)	97	3.85	38	3.19	59	4.46
moderately severe (15-19)	38	1.51	16	1.34	22	1.66
severe (20-27)	18	0.71	7	0.59	10	0.76

### 3.4 Internal consistency

Cronbach’s alpha of the PHQ-9 for the full sample was *α* = 0.90, 95% CI [0.89, 0.91]. McDonald’s omega of the PHQ-9 for the full sample was *ω* = 0.93, 95% CI [0.92, 0.94].

### 3.5 Factorial validity

A CFA was conducted to assess the unidimensional structure of the PHQ-9. The fit indices indicated reasonable model fit, with a robust CFI of 0.91, a robust TLI of 0.89, and an SRMR of 0.044. However, the robust RMSEA was 0.17 (90% CI [0.153, 0.186]), suggesting some misfit.

To improve the model, we inspected modification indices and introduced residual correlations between items with overlapping content: #1 (Little interest or pleasure) with #2 (Down, depressed, hopeless) and #3 (Sleep problems) with #4 (Tired, little energy). These adjustments improved the fit indices (CFI = 0.96, RMSEA = 0.11), as detailed in **Supplementary Table C2**.

Despite these modifications, factor score correlations between the original and adjusted models remained high (r > 0.999), confirming the stability of the latent structure and supporting the unidimensionality of the PHQ-9. Strong standardized factor loadings (0.79–0.89) further reinforced this. A SEM path diagram can be found in **Supplementary Figure C2** of the **Supplementary Materials**.

### 3.6 Measurement invariance

The fit measures obtained in the measurement invariance analyses of the PHQ-9 are presented in **Supplementary Table C4** in the **Supplementary Materials**. Adequate CFI and RMSEA differences were found for all invariance steps and groups. The sensitivity analyses using MLR estimation confirmed measurement invariance regarding age, gender and education. Given the very small CFA and RMSEA differences in the original analysis using WLSMV estimation, measurement invariance regarding age × gender and income is likely, yet inconclusive.

## 4 Discussion

The present study investigates the psychometric quality of the PHQ-9 using a large and representative sample of the German general population. Based on coefficient *ω*, the PHQ-9 can be attested a high internal consistency. Furthermore, the analyses showed comparable factor structures using MGCFA in the subgroups that were compared (gender, age groups). The overall factor structure assumed for the PHQ-9 fitted well for the defined gender and age groups thus indicating that the PHQ-9 can be used for gender or age comparisons. Lastly, the reported correlation between the PHQ-9 and the GAD-7 as well as the BSI-18 lies within the range of previous studies. Overall, the present results are in line with results of previous normative studies (19), suggesting that the PHQ-9 is an efficient, reliable, and valid instrument for assessing depressive symptoms. We provide updated norm tables for clinical practice, which was the main aim of this study. These percentiles are tabulated (see **Table 2** and **Supplementary Tables C6**, **C7**) for different age ranges and available both gender-specific and gender-unspecific. On the population level, the suggested clinical cut-off of 10 points for the PHQ-9 falls in the range of percentiles 92-98, which reflects above average to well above average values.

The percentiles obtained in the present study are comparable to values reported by previous work in the German general population (19). It is however noteworthy that our norms are almost identical to previous norms “+1” i.e. our percentiles closely align to those of (19) but with a shift of almost 1 point on the PHQ-Sum score. Our values also closely align with more recent percentile-ranks provided by Shin et al. (16) based on general population data from Korea as well as data from Tomitaka et al. (17) from the US general population. (For a more detailed comparison see **Supplementary Figure C3** in the online supplements).

The high share of participants indicating virtually no depressive symptoms (41%) might seem counter intuitive given the ongoing COVID-19 pandemic during the survey period. However, a decrease of mood disorder symptoms is in line with other findings from large representative German studies indicating, for example, that contrary to common belief levels of domestic violence (38) decreased and overall mental health increased [e.g., (39)]. There are several large surveys which include the PHQ-9 that were carried out in the German general population [e.g., (40, 41)] as well as in other western general populations [e.g., (12–15, 42)]. While the depressive symptom burden varies considerably among these studies they all report significantly higher levels of depression in the general population than the study at hand. All of these studies utilized large online convenience samples and the surveys were framed as assessing pandemic related mental health burden. We consider these differences in study design to be crucial with respect to self-selection bias and hence believe that our results based on a face-to-face survey using a representative sample is an important contribution, contrasting and potentially balancing the current scientific discourse. Furthermore, these findings highlight the necessity for frequently updated norms and warns against the assumptions of stable prevalence rates and symptom burden. Additionally, our norms serve as an important reference point for longitudinal studies and provide an indication of symptom variability over time on the population level.

### 4.1 Limitations

Despite the current study being based on high quality data from a large representative sample it is not without limitations. First, the response rate was only 42.6%. However, general population studies commonly have significantly lower response rates than clinical studies and the response rate of this study is comparable to similar surveys [e.g., (31, 43, 44)]. Despite significant efforts to maximize sample representativeness, some degree of non-response remains unavoidable with the current design and there is a potential for bias due to non-response. Unfortunately, the possibility of non-response bias cannot be systematically assessed as no demographic information of non-responders is available. This would only be possible if sampling were based on registry data which is not accessible without government permit in Germany. Furthermore, the current study does not allow any conclusions regarding the diagnostic efficiency of the PHQ-9 as no clinical interviews were conducted. While the presented norms and psychometric properties can serve as valuable reference data for clinical research the generalizability to clinical samples is limited.

### 4.2 Conclusion

In summary, the German version of the PHQ-9 has shown sound psychometric properties in a large representative population sample. While the percentiles for the sum-score are comparable to similar studies in community samples, the change in symptom burden compared to previous German norm values from over a decade earlier make the present study an important reference. Furthermore, the results from the present study serve as a notable counter point in the interpretation of COVID-19 related findings including the PHQ-9. We suggest updating the norms again in the near future to gain a deeper understanding whether the present findings have to be interpreted as pandemic related or as “the new normal”.

## Data Availability

The raw data supporting the conclusions of this article will be made available by the authors, without undue reservation.

## Footnotes

1 Kliem S, Sachser C, Lohmann A, Baier D, Brähler E, Fegert J, et al. Psychometric evaluation and community norms of the GAD-7, based on a representative German sample. (2024).

## Supplementary material

Supplementary Figure C1 MGCFA models for Measurement Invariance Analysis.

Supplementary Figure C2 One factor CFA model of the PHQ-9.

Supplementary Figure C3 One factor CFA model of the PHQ-9 with residual correlation #1 #2.

Supplementary Figure C4 Figure C4. One factor CFA model of the PHQ-9 with residual correlation #1 #2 and #3 #4.

Supplementary Figure C5 Comparison of PHQ-9 Sum-scores across community studies.

Supplementary Table A1 Comparison Sample Distribution vs Population Distribution for Age and Gender.

Supplementary Table A2 Comparison Sample Distribution vs Population Distribution for Population of Federal States.

Supplementary Table A3 Demographic characteristics of the study sample at hand.

Supplementary Table A4 Demographic characteristics of a comparable survey with identical sampling procedure. Item-wise missingness of PHQ-Items. Item-wise Missingness of GAD-Items. Item-wise Missingness of BSI-18 Items.

Supplementary Table C1 Parameter Constraints for MGCFA.

Supplementary Table C2 Fit indices of One Factor Model and Alternative models with additional residual correlations.

Supplementary Table C3 Factor score correlations: PHQ-9 sum score, One Factor Model, Alternative models with additional residual correlations.

Supplementary Table C4 Results of measurement invariance analyses.

Supplementary Table C5 Means (M), standard deviation (SD), and group differences for the PHQ-9 items.

Supplementary Table C6 Means, standard deviations, and correlations with confidence intervals PHQ-9 Items.

Supplementary Table C7 Scale correlations: PHQ-9, GAD-7, BSI-18.

Supplementary Table C8 Population based norms of the PHQ-9 (male subsample).

Supplementary Table C9 Population based norms of the PHQ-9 (female subsample).

Supplementary Table D1 Means (M), standard deviation (SD), and group differences for the PHQ-9 items (unimputed data).

Supplementary Table D2 Scale correlations: PHQ-9, GAD-7, BSI-18 (unimputed data).

Supplementary Table D3 Population based norms of the PHQ-9 (total sample – unimputed data).

Supplementary Table E1 Results of measurement invariance analyses (MLR estimator).
