# Supplementary material for: Psychometric evaluation and community norms of the PHQ-9, based on a representative German sample
Source: Front Psychiatry. 2024 Dec 12;15:1483782. doi: 10.3389/fpsyt.2024.1483782 (PMC11670475; doi:10.3389/fpsyt.2024.1483782)
Supplement: Supplementary Figure C1 — MGCFA models for Measurement Invariance Analysis. [file DataSheet1.pdf]

Supplemental Materials

SECTION A SAMPLING PROCEDURE, COVID MEASURES AND REPRESENTATIVENESS

Section A provides further background information on the sampling procedure and sample composition. As the survey was conducted during the COVID 19 pandemic we provide the reader with additional information on sampling, COVID measures, regional distribution of participants as well as comparison data for demographic characteristics. All of this additional information should serve as indications of the samples representativeness and allow for the investigation of concerns regarding pandemic related biases.

Sampling Procedure Using the ADM Sampling System for Face-to-Face Interviews

In Germany, there is no publicly accessible register of private households or individuals, as municipal resident directories are only available for surveys when there is a claim of public interest. The ADM Sampling System for face-to-face interviews (F2F), managed by the ADM Sampling Working Group (of which USUMA is a member), addresses this gap by providing a sampling framework that allows for the creation of representative samples of private households and the individuals living in them.

Overview of the ADM Sampling System F2F:

Area-Based Sampling: The ADM system is an area-based sampling method covering all inhabited areas in Germany. It relies on municipal boundaries, intra-municipal subdivisions, statistical data, and electronic regional divisions created for navigation systems. Approximately 53,000 geographic areas are electronically defined, each containing at least 350 and an average of 700 households.

Stratification and Selection: These areas are first stratified by districts and BIK types (a classification system for urban and rural areas), creating around 1,500 regional strata. A total of 128 sampling networks, consisting of 210 selection areas in the old federal states (West Germany) and 48 in the new federal states (East Germany), are drawn proportionally to household distribution using a random allocation method developed by L.H. Cox. These networks are non-overlapping and can be combined due to the Cox allocation method.

Household and Individual Selection:

1. *Household Selection*: In the second stage, within each selected geographic area, households are chosen using the random-route method. A starting address (Sample-Point) and a step size for systematic random selection are provided. Interviewers list every third household starting from the Sample-Point until 23 valid private household addresses per Sample-Point are identified. For the study, 258 Sample-Points were used, resulting in the selection of 5,676 households.

2. *Individual Selection:* In the final stage, the target individual within each selected household is chosen using the "Swedish key" (a random selection method ensuring unbiased person selection).

The ADM F2F sampling method ensures that both the area selection and the household and individual selection processes follow random sampling principles, resulting in representative probability samples that conform to statistical random models.

COVID Measures during the Survey

The data collection phase for this study commenced at a time when the COVID-19 pandemic had already started to spread within Germany. Despite initial concerns, fieldwork began as planned on December 14, 2020, as the full extent of the pandemic's impact on the German population was not yet apparent at that time. To ensure the safety of both participants and field staff, comprehensive hygiene protocols were implemented during data collection.

In preparation for fieldwork, all personnel involved in data collection were provided with updated guidelines and special instructions in the fall of 2020. These included written hygiene regulations tailored for conducting interviews during the pandemic. Specific instructions were provided for contact initiation at participants' homes, including adherence to social distancing guidelines. Interviewers were also equipped with appropriate personal protective equipment, including face masks, to minimize the risk of virus transmission during face-to-face interactions.

These measures were essential in maintaining representativeness during the pandemic, as they allowed us to proceed with the survey despite the challenging circumstances. While the COVID-19 pandemic did present potential challenges for participation, these protocols helped mitigate risks and ensure that a broad cross-section of the German population could still be reached.

Although response rates may have been influenced by health concerns related to the pandemic, particularly among vulnerable populations such as older adults or individuals with pre-existing health conditions, the implemented safety measures ensured that participation was as representative as possible under the circumstances. Additionally, these precautions helped alleviate concerns of participants regarding face-to-face interactions, which could have otherwise led to higher levels of non-response.

The following tables compare the sample distribution with the population distribution regarding age, gender and residency in federal state based on data from the Federal Statistical Office (Statistisches Bundeamt).

Table A1
Comparison Sample Distribution vs Population Distribution for Age and Gender

**Age Group**	**Gender**	**Sample Distribution**	**Population Distribution**
		%	%
16-19	male	1.6	2.4
16-19	female	1.9	3.2
20-29	male	5.8	7.1
20-29	female	6.5	6.5
30-39	male	7.3	7.6
30-39	female	7.7	7.3
40-49	male	7.2	7.3
40-49	female	8.7	7.2
50-59	male	9.7	9.4
50-59	female	10.2	9.4
60-69	male	8.8	7.0
60-69	female	8.0	7.4
70+	male	6.9	7.7
70+	female	9.6	10.5

Table A2
Comparison Sample Distribution vs Population Distribution for Population of Federal States

Federal State	**Sample Distribution**	**Population Distribution**
	%	%
Schleswig-Holstein	3.9	3.5
Hamburg	2.4	2.2
Lower Saxony	9.6	9.6
Bremen	0.8	0.8
North Rhine-Westphalia	21.3	21.5
Hesse	6.6	7.5
Rhineland-Palatinate	4.4	4.9
Baden-Württemberg	11.7	13.3
Bavaria	14.3	15.7
Saarland	1.0	1.2
Berlin-West	3.5	2.4
Brandenburg	3.8	3.0
Mecklenburg-Western Pomerania	2.4	2.0
Saxony	6.6	4.9
Saxony-Anhalt	2.5	2.7
Thuringia	3.0	2.6
Berlin-East	2.3	2.0

In the following section we provide demographic information on our sample as well as a sample from 2013 reported in Kliem et al 2015. The reference survey was conducted pre COVID and used the same sampling procedure. This information is provided to allow the reader a better understanding whether pandemic conditions might have influenced the sample composition.

Table A3
Demographic characteristics of the study sample at hand

	Male (*N* = 1193)	Female (*N* = 1322)	Diverse (*N* = 4)	Total (*N* = 2519)
Age in Years				
Mean (SD)	50.1 (17.7)	50.5 (18.3)	44.8 (26.5)	50.3 (18.1)
Median [Min, Max]	52.0 [16, 96]	51.0 [16, 96]	41.5 [21, 75]	51.0 [16, 96]
Age Categories				
14-24	102 (8.5%)	125 (9.5%)	2 (50.0%)	229 (9.1%)
25-34	190 (15.9%)	174 (13.2%)	0 (0.0%)	364 (14.5%)
35-44	174 (14.6%)	225 (17.0%)	0 (0.0%)	399 (15.8%)
45-54	195 (16.3%)	216 (16.3%)	0 (0.0%)	411 (16.3%)
55-64	244 (20.5%)	243 (18.4%)	1 (25.0%)	488 (19.4%)
65-74	190 (15.9%)	200 (15.1%)	0 (0.0%)	390 (15.5%)
75+	98 (8.2%)	139 (10.5%)	1 (25.0%)	238 (9.4%)
Living with partner				
Living together	729 (61.1%)	737 (55.7%)	2 (50.0%)	1468 (58.3%)
Not living together	444 (37.2%)	565 (42.7%)	2 (50.0%)	1011 (40.1%)
Missing	20 (1.7%)	20 (1.5%)	0 (0.0%)	40 (1.6%)
Education				
≤ 8 years	358 (30.0%)	378 (28.6%)	0 (0.0%)	736 (29.2%)
9-11 years	538 (45.1%)	622 (47.1%)	3 (75.0%)	1163 (46.2%)
≥ 12 years	262 (22.0%)	288 (21.8%)	1 (25.0%)	551 (21.9%)
Current student	25 (2.1%)	22 (1.7%)	0 (0.0%)	47 (1.9%)
Missing	10 (0.8%)	12 (0.9%)	0 (0.0%)	22 (0.9%)
Employment Status				
Pupil/Student/In training	65 (5.4%)	88 (6.7%)	1 (25.0%)	154 (6.1%)
Employed	691 (57.9%)	658 (49.8%)	2 (50.0%)	1351 (53.6%)
Unemployed	94 (7.9%)	112 (8.5%)	0 (0.0%)	206 (8.2%)
Homemaker	4 (0.3%)	46 (3.5%)	0 (0.0%)	50 (2.0%)
Retired	334 (28.0%)	390 (29.5%)	1 (25.0%)	725 (28.8%)
Missing	5 (0.4%)	28 (2.1%)	0 (0.0%)	33 (1.3%)
Household income				
< 1250 €/month	158 (13.2%)	224 (16.9%)	1 (25.0%)	383 (15.2%)
1250 - < 2500 €/month	438 (36.7%)	536 (40.5%)	1 (25.0%)	975 (38.7%)
≥ 2500 €/month	597 (50.0%)	562 (42.5%)	2 (50.0%)	1161 (46.1%)

Table A4
Demographic characteristics of a comparable survey with identical sampling procedure

	Male (*N* = 1174)	Female (*N* = 1334)	Total (*N* = 2508)
Age (years)			
Mean (SD)	49.2 (18.2)	50.1 (18.4)	49.7 (18.3)
Median [Min, Max]	50.0 [14, 92]	50.0 [14, 92]	50.0 [14, 92]
Age Categories			
14-24	134 (11.4%)	123 (9.2%)	257 (10.2%)
25-34	152 (12.9%)	208 (15.6%)	360 (14.4%)
35-44	180 (15.3%)	202 (15.1%)	382 (15.2%)
45-54	213 (18.1%)	232 (17.4%)	445 (17.7%)
55-64	225 (19.2%)	229 (17.2%)	454 (18.1%)
65-74	177 (15.1%)	204 (15.3%)	381 (15.2%)
75+	93 (7.9%)	136 (10.2%)	229 (9.1%)
Living with partner			
Living together	663 (56.5%)	652 (48.9%)	1315 (52.4%)
Not living together	511 (43.5%)	682 (51.1%)	1193 (47.6%)
Education			
≤ 8 years	432 (36.8%)	510 (38.2%)	942 (37.6%)
9-11 years	453 (38.6%)	570 (42.7%)	1023 (40.8%)
≥ 12 years	238 (20.3%)	217 (16.3%)	455 (18.1%)
Current student	45 (3.8%)	33 (2.5%)	78 (3.1%)
Missing	6 (0.5%)	4 (0.3%)	10 (0.4%)
Employment Status			
Pupil/Student	103 (8.8%)	89 (6.7%)	192 (7.7%)
Employed	651 (55.5%)	608 (45.6%)	1259 (50.2%)
Unemployed	85 (7.2%)	104 (7.8%)	189 (7.5%)
Homemaker	4 (0.3%)	100 (7.5%)	104 (4.1%)
Retired	329 (28.0%)	416 (31.2%)	745 (29.7%)
Missing	2 (0.2%)	17 (1.3%)	19 (0.8%)
Household income			
< 1250 €/month	197 (16.8%)	320 (24.0%)	517 (20.6%)
1250 - < 2500 €/month	527 (44.9%)	619 (46.4%)	1146 (45.7%)
≥ 2500 €/month	417 (35.5%)	352 (26.4%)	769 (30.7%)
Missing	33 (2.8%)	43 (3.2%)	76 (3.0%)

SECTION B. MISSING DATA

Section B contains additional results. Due to the paper being a short report the number of figures and tables allowed in the main body of the manuscript are limited. We therefore provide them here.

Item-wise missingness of PHQ-Items

#1: 0.32 %	#2: 0.52 %	#3: 0.40%	#4: 0.40 %	#5: 0.67 %
#6: 0.56 %	#7: 0.44 %	#8: 0.52 %	#9: 0.56 %	

Item-wise Missingness of GAD-Items

#1: 0.32 %	#2: 0.56 %	#3: 0.40 %	#4: 0.71 %	#5: 0.44 %
#6: 0.40 %	#7: 0.40 %			

Item-wise Missingness of BSI-18 Items

#1: 0.36 %	#2: 0.36 %	#3: 0.40 %	#4: 0.36 %	#5: 0.48 %
#6: 0.44 %	#7: 0.6 %	#8: 0.44 %	#9: 0.48 %	#10: 0.44 %
#11 0.36 %	#12 0.36 %	#13: 0.48 %	#14: 0.36 %	#15: 0.40 %
#16: 0.40 %	#17 0.40 %	#18 0.40 %		

SECTION C. ADDITIONAL RESULTS

Section C contains additional results. Due to the paper being a short report the number of figures and tables allowed in the main body of the manuscript are limited. We therefore provide them here.

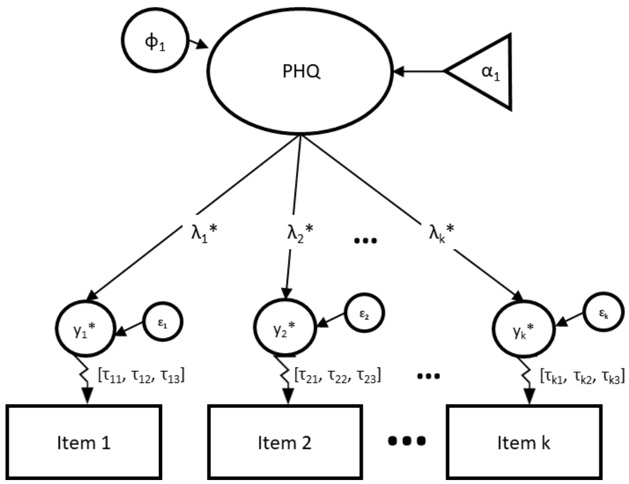

Figure C1. MGCFA models for Measument Invariance Analysis:

(1) Configural invariance: Same CFA model fit in all groups;

(2) Threshold Invariance: thresholds $\tau_{1i}...\tau_{4i}$ constrained to be equal across groups;

(3) Metric Invariance: additionally factor loadings λ_i constrained to be equal across groups;

(4) Scalar invariance: additionally intercepts constrained to be equal across groups;

(5) Residual invariance: additionally residual variances ϵ_i constrained to be equal across groups

Table C1. Parameter Constraints for MGCFA

Parameters	Threshold invariance `group.equal = “thresholds”*`	Metric Invariance group.equal = `c(“thresholds, “loadings”)*`	Scalar Invariance group.equal = `c(“theresholds”, “loadings”, “intercepts”)*`	Residual Invariance group.equal = `c(“thresholds”, “loadings”, “intercepts”)*`
Item loadings	free	equal across groups	equal across groups	equal across groups
Intercepts				
Items	constrained to 0 in first group	constrained to 0 in first group	equal (0) across groups	equal (0) across groups
Latent	constrained to 0 in all groups	constrained to 0 in all groups	constrained to zero in first group	constrained to zero in first group
Thresholds	equal across groups	equal across groups	equal across groups	equal across groups
Variances				
Residual	constrained to 1 in first group	constrained to 1 in first group	constrained to 1 in first group	equal (1) across groups
Latent	constrained to 1 in all groups	constrained to 1 in first group	constrained to 1 in first group	constrained to 1 in first group

Note: *setting for call of the `measEq.syntax()` function from the `semTools` package

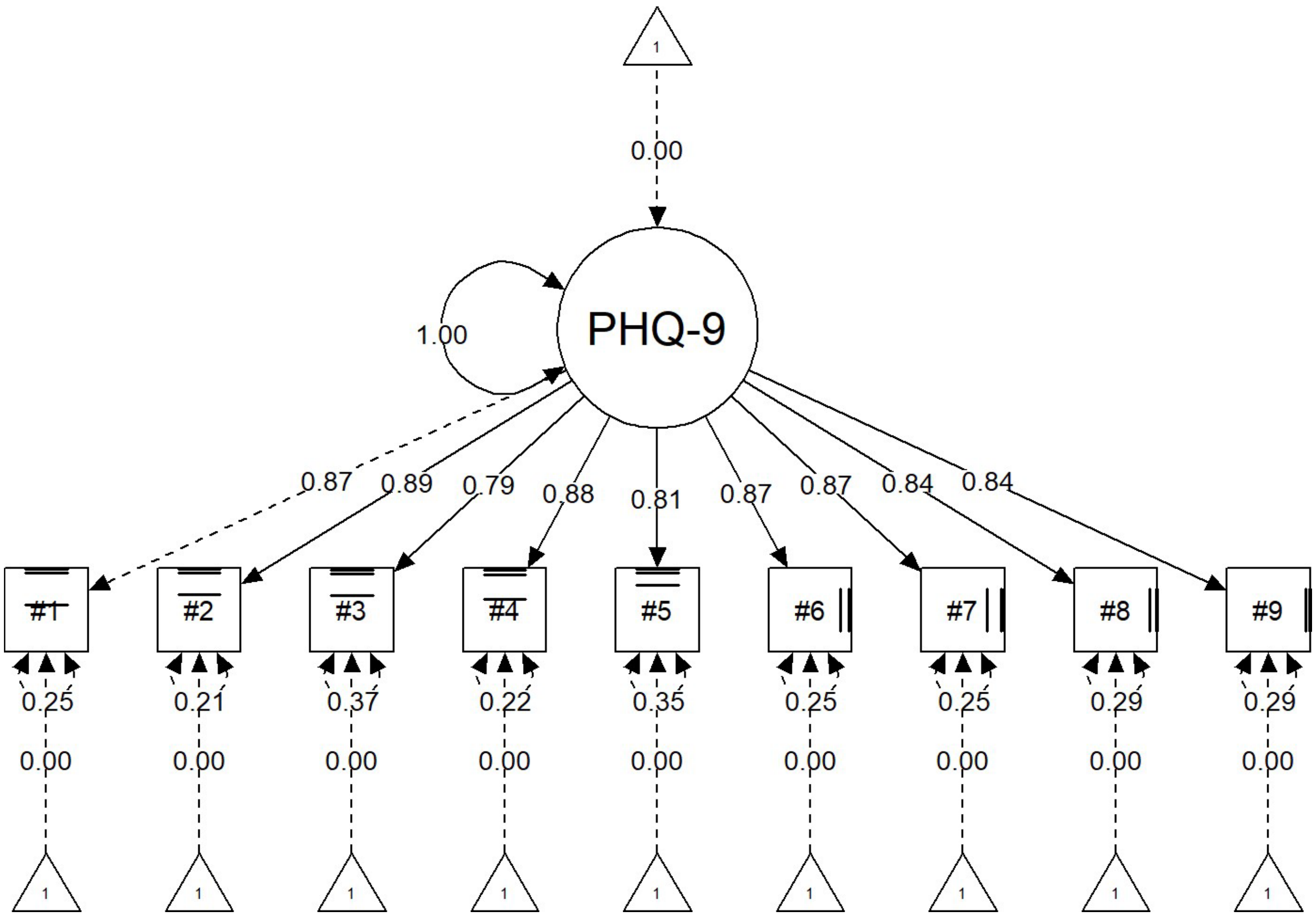

Figure C2. One factor CFA model of the PHQ-9.

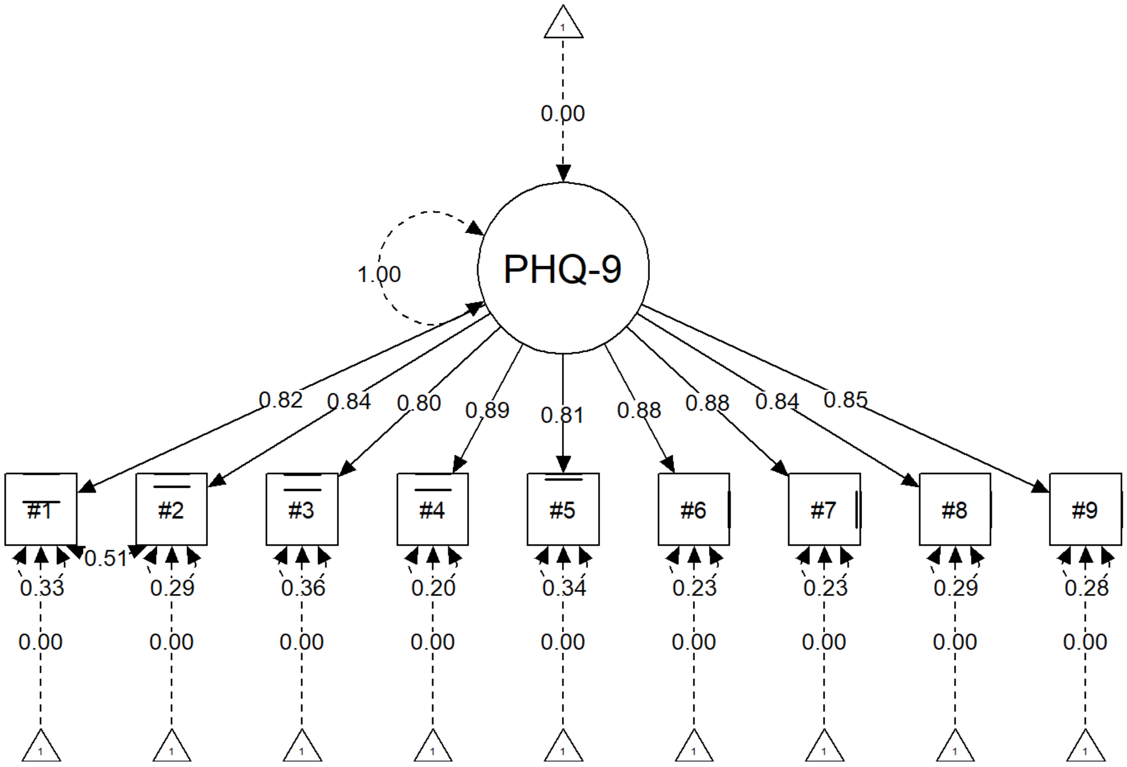

Figure C3. One factor CFA model of the PHQ-9 with residual correlation #1~~#2.

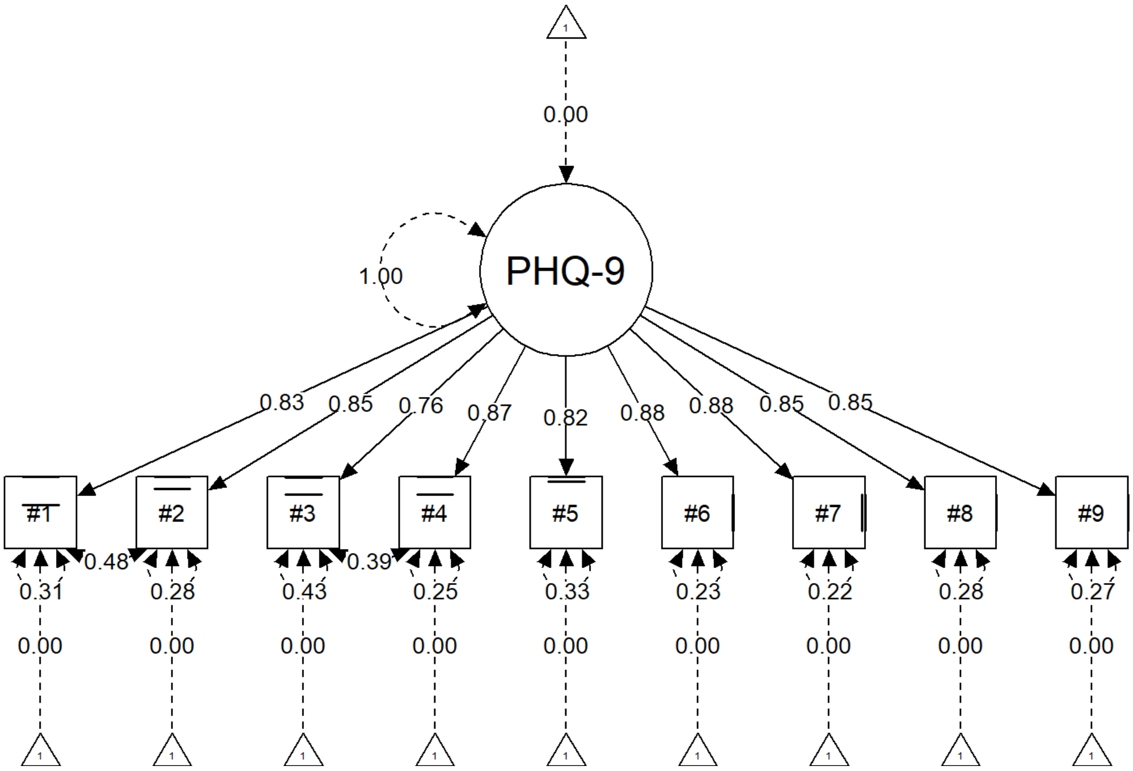

Figure C4. One factor CFA model of the PHQ-9 with residual correlation #1~~#2 and #3~~#4.

Table C2

Fit indices of One Factor Model and Alternative models with additional residual correlations

Model	χ^2	df	CFI	TLI	SRMR	RMSEA [90% CI]
One Factor Model	256.81	27	0.91	0.88	0.04	0.17 [0.15, 0.19]
Model with residual correlation #1~~#2	143.91	26	0.94	0.92	0.04	0.14 [0.12, 0.16]
Model with residual correlations #1~~#2 and #3~~#4	85.74	25	0.96	0.95	0.03	0.11 [0.09, 0.13]

Note. χ^2 = chi-square; *df* = degrees of freedom; CFI = robust comparative fit index; TLI = robust Tucker-Lewis index; SRMR = standardized root mean square residual; RMSEA = robust root mean square error of approximation; CI = confidence interval.

Table C3

Factor score correlations: PHQ-9 sum score, One Factor Model, Alternative models with additional residual correlations

Variable	M	SD	1	2	3
1. PHQ-9 Sum-score	2.6888	3.8663			
2. Factor-score Onefactor	0.1130	0.7912	0.9386 ** (0.9338, 0.9431)		
3. Factor-score Mod1	0.1018	0.8037	0.9370 ** (0.932, 0.9416)	0.9990 ** (0.9989, 0.9991)	
4. Factor-score Mod2	0.0949	0.8111	0.9357 ** (0.9306, 0.9404)	0.9994 ** (0.9993, 0.9994)	0.9996 ** (0.9996, 0.9996)

Note. PHQ-9 = Patient Health Questionnaire; Mod1 = incl. residual correlation #1~~#2; Mod2 = incl. residual correlation #1~~#2 and #3~~#4; ** indicates $p < .001$.

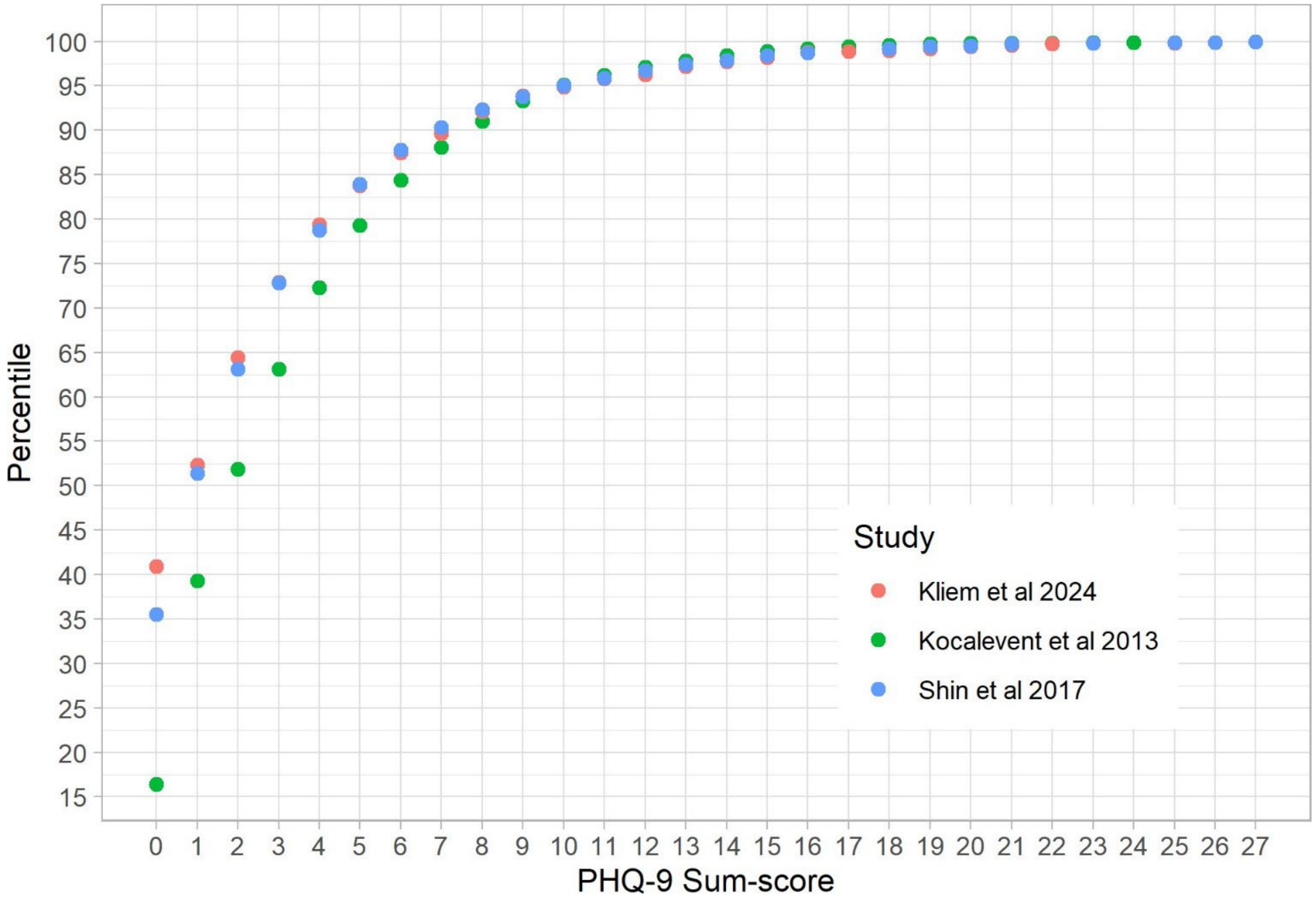

Figure C5. Comparison of PHQ-9 Sum-scores across community studies.
The x-axis shows the PHQ-9 scores and the y-axis the population norms (as percentiles) for three different studies conducted on representative community samples. While the percentiles of Shin et al and the present study are highly similar, the study by Kocalevent et al shows a one point "shift" up to a sum-score of 10.

Table C4

Results of measurement invariance analyses

	χ^2	df	CFI	ΔCFI	RMSEA	ΔRMSEA	Measurement invariance
Gender (male, female)							
Configural invariance	260.329	54	0.996	-	0.079	-	-
Threshold invariance [1]	262.962	63	0.996	0	0.073	-0.006	✓
Metric invariance [2]	271.515	71	0.996	0	0.065	-0.007	✓
Scalar invariance [3]	307.296	79	0.996	0	0.061	-0.004	✓
Full invariance [4]	337.353	88	0.996	0	0.055	-0.006	✓
Age (<= 51, >51)							
Configural invariance	283.442	54	0.996	-	0.082	-	-
Threshold invariance [1]	291.267	63	0.996	0	0.076	-0.006	✓
Metric invariance [2]	305.064	71	0.996	0	0.069	-0.007	✓
Scalar invariance [3]	327.006	79	0.996	0	0.063	-0.006	✓
Full invariance [4]	344.519	88	0.995	0	0.056	-0.007	✓
Age * Gender							
Configural invariance	302.796	108	0.997	-	0.078	-	-
Threshold invariance [1]	315.421	132	0.997	0	0.071	-0.008	✓
Metric invariance [2]	341.825	156	0.997	0	0.062	-0.009	✓

Table C4 continued

	χ^2	df	CFI	ΔCFI	RMSEA	ΔRMSEA	Measurement invariance
Scalar invariance [3]	414.013	180	0.996	-0.001	0.057	-0.004	✓
Full invariance [4]	474.541	207	0.995	-0.001	0.051	-0.006	✓
Income (<= 1750, >1750)							
Configural invariance	282.78	54	0.996	-	0.082	-	-
Threshold invariance [1]	285.077	63	0.996	0	0.075	-0.007	✓
Metric invariance [2]	290.857	71	0.996	0	0.067	-0.008	✓
Scalar invariance [3]	317.694	79	0.996	0	0.061	-0.006	✓
Full invariance [4]	338.306	88	0.996	0	0.055	-0.007	✓
Educational attainment (no university entry qualification, university entry qualification)							
Configural invariance	285.170	54	0.996	-	0.079	-	-
Threshold invariance [1]	288.026	62	0.996	0	0.074	-0.005	✓
Metric invariance [2]	298.055	70	0.996	0	0.068	-0.006	✓
Scalar invariance [3]	307.682	78	0.996	0	0.059	-0.008	✓
Full invariance [4]	321.422	87	0.996	0	0.053	-0.006	✓

Note. All fit statistics are robust; CFI = Comparative Fit Index; CFI = CFI-differences for the different measurement invariance levels; RMSEA = Root Mean Square Error of Approximation; RMSEA = RMSEA- differences for the different measurement invariance levels; ✓= ΔCFI < -.010 complemented by RMSEA ≥ .015 indicates a violation of measurement invariance; marks measurement invariance for the respective level; [1] equivalency of thresholds; [2] equivalency of thresholds + factor loadings; [3] equivalency of thresholds + factor loadings + equivalency of constants; [4] equivalency of thresholds + factor loadings + equivalency of constants + unique-factor variances.

Table C5

Means (M), standard deviation (SD), and group differences for the PHQ-9 items

	Total		Male		Female		Group differences			
	M	SD	M	SD	M	SD	d [95% CI]	t	df	p
PHQ-9	2.69	3.87	2.38	3.68	2.95	3.98	-0.15 [-0.23,-0.07]	-3.66	2513	<0.001
Little interest or pleasure	0.51	0.65	0.48	0.64	0.54	0.66	-0.09 [-0.17,-0.01]	-2.55	2513	0.011
Down/ depressed/ hopeless	0.38	0.63	0.33	0.59	0.42	0.65	-0.14 [-0.22,-0.07]	-3.61	2513	<0.001
Sleep problems	0.42	0.68	0.35	0.62	0.47	0.72	-0.18 [-0.26,-0.1]	-4.58	2513	<0.001
Tired/ little energy	0.47	0.70	0.39	0.65	0.53	0.73	-0.2 [-0.28,-0.12]	-5.11	2513	<0.001
Poor appetite/ overeating	0.26	0.58	0.23	0.55	0.29	0.60	-0.1 [-0.18,-0.03]	-2.75	2513	0.006
Feeling bad about self/failure	0.19	0.52	0.19	0.51	0.19	0.53	0 [-0.08,0.08]	-0.33	2513	0.744
Trouble concentrating	0.27	0.58	0.24	0.55	0.29	0.59	-0.09 [-0.17,-0.01]	-2.06	2513	0.039
Moving/speaking slowly - Fidgety/ restless	0.13	0.42	0.12	0.42	0.14	0.42	-0.05 [-0.13,0.03]	-1.12	2513	0.264
Suicidal ideation/ self-injury	0.07	0.30	0.07	0.30	0.07	0.30	0 [-0.08,0.08]	-0.33	2513	0.742

Note. PHQ = Patient Health Questionnaire

Table C6

Means, standard deviations, and correlations with confidence intervals PHQ-9 Items

Variable	M	SD	1	2	3	4	5	6	7	8
1. #1	0.51	0.65								
2. #2	0.38	0.63	.70**<br>[.68, .72]							
3. #3	0.42	0.68	.52**<br>[.49, .54]	.51**<br>[.48, .54]						
4. #4	0.47	0.70	.62**<br>[.60, .65]	.62**<br>[.60, .65]	.66**<br>[.63, .68]					
5. #5	0.26	0.58	.49**<br>[.46, .52]	.52**<br>[.49, .55]	.48**<br>[.45, .51]	.58**<br>[.55, .61]				
6. #6	0.19	0.52	.51**<br>[.48, .54]	.57**<br>[.54, .60]	.45**<br>[.42, .48]	.51**<br>[.48, .55]	.53**<br>[.51, .56]			
7. #7	0.27	0.58	.54**<br>[.51, .57]	.58**<br>[.55, .60]	.52**<br>[.49, .55]	.59**<br>[.57, .62]	.55**<br>[.52, .58]	.63**<br>[.60, .65]		
8. #8	0.13	0.42	.39**<br>[.36, .43]	.44**<br>[.41, .47]	.40**<br>[.37, .44]	.44**<br>[.41, .47]	.47**<br>[.44, .50]	.55**<br>[.53, .58]	.58**<br>[.55, .61]	
9. #9	0.07	0.30	.37**<br>[.34, .41]	.43**<br>[.40, .46]	.37**<br>[.34, .41]	.40**<br>[.37, .43]	.42**<br>[.39, .45]	.50**<br>[.47, .53]	.47**<br>[.43, .50]	.56**<br>[.53, .58]

Note. Note. M and SD are used to represent mean and standard deviation, respectively. Values in square brackets indicate the 95% confidence interval. The confidence interval is a plausible range of population correlations that could have caused the sample correlation (Cumming, 2014). * indicates p < .05. ** indicates p < .01.

Table C7

Scale correlations: PHQ-9, GAD-7, BSI-18

Variable	M	SD	1	2	3	4	5
1. PHQ-9	2.69	3.87					
2. GAD-7	2.17	3.26	.85**<br>[.84, .86]				
3. BSI GSI	4.36	7.63	.79**<br>[.77, .80]	.76**<br>[.74, .77]			
4. BSI Somatization	1.21	2.54	.64**<br>[.62, .67]	.58**<br>[.55, .60]	.87**<br>[.86, .88]		
5. BSI Anxiety	1.26	2.55	.70**<br>[.68, .72]	.72**<br>[.70, .74]	.91**<br>[.91, .92]	.73**<br>[.71, .75]	
6. BSI Depression	1.89	3.38	.77**<br>[.75, .78]	.73**<br>[.71, .75]	.92**<br>[.91, .92]	.66**<br>[.64, .68]	.76**<br>[.74, .77]

Note. PHQ-9 = Patient Health Questionnaire; GAD-7 = Generalized Anxiety scale; BSI GSI = Brief Symptom Inventory Global Severity Index; BSI Somatization = Brief Symptom Inventory Somatization Subscale; BSI Anxiety = Brief Symptom Inventory Anxiety Subscale; BSI Depression = Brief Symptom Inventory Depression Subscale; * indicates $p < .05$., ** indicates $p < .001$.

Table C8

Population based norms of the PHQ-9 (male subsample)

PHQ-9	Total	age 16-24	age 25-34	age 35-44	age 45-54	age 55-64	age 65-74	age 75+
0	44.9	46.1	56.8	46.0	41.0	43.4	40.0	39.8
1	56.7	56.9	67.4	66.7	52.8	53.7	49.5	46.9
2	68.1	65.7	80.0	78.2	61.5	65.2	65.3	56.1
3	76.2	76.5	84.7	85.6	70.3	72.1	75.8	65.3
4	82.2	82.4	89.5	89.7	74.9	79.1	82.6	76.5
5	86.5	87.3	92.1	90.8	81.5	83.6	87.4	82.7
6	89.8	92.2	95.3	92.5	87.7	86.5	88.4	86.7
7	91.5	95.1	95.8	93.7	89.2	88.5	90.5	89.8
8	93.7	96.1	95.8	94.8	92.3	92.6	93.7	90.8
9	94.8	98.0	95.8	96.0	93.8	93.9	95.3	90.8
10	95.6	98.0	97.9	96.0	94.9	94.3	95.8	91.8
11	96.4	98.0	98.9	96.6	94.9	95.9	96.8	92.9
12	96.8	98.0	> 99.9	96.6	95.4	95.9	97.4	93.9
13	97.5	98.0	> 99.9	97.1	97.9	96.3	97.4	94.9
14	98.0	99.0	> 99.9	97.7	98.5	96.3	98.4	95.9
15	98.5	99.0	> 99.9	98.3	99.0	97.1	98.9	96.9
16	99.0	99.0	> 99.9	98.9	99.0	98.4	99.5	98.0
18	99.1	99.0	> 99.9	98.9	99.0	98.4	99.5	99.0
19	99.3	99.0	> 99.9	98.9	99.5	98.8	99.5	> 99.9
20	99.4	> 99.9	> 99.9	98.9	99.5	98.8	99.5	> 99.9
21	99.6	> 99.9	> 99.9	99.4	> 99.9	98.8	99.5	> 99.9
23	99.7	> 99.9	> 99.9	> 99.9	> 99.9	98.8	99.5	> 99.9
25	99.8	> 99.9	> 99.9	> 99.9	> 99.9	99.2	> 99.9	> 99.9
27	> 99.9	> 99.9	> 99.9	> 99.9	> 99.9	> 99.9	> 99.9	> 99.9

Table C9

Population based norms of the PHQ-9 (female subsample)

PHQ-9	Total	age 16-24	age 25-34	age 35-44	age 45-54	age 55-64	age 65-74	age 75+
0	37.4	38.4	42.5	43.6	37.5	36.2	36.5	23.7
1	48.5	46.4	54.0	55.6	52.3	45.7	44.0	37.4
2	61.1	56.0	64.4	68.9	64.4	57.6	56.5	56.8
3	70.1	64.8	69.5	75.1	74.1	68.3	68.5	66.9
4	76.9	68.8	77.6	79.1	82.9	73.7	77.5	74.8
5	81.5	76.8	81.0	80.9	86.6	79.4	82.5	82.0
6	85.6	81.6	85.6	85.3	89.8	83.1	86.0	86.3
7	88.0	84.0	87.9	87.1	91.2	88.5	88.0	87.8
8	90.8	88.8	91.4	88.4	93.5	89.7	93.0	89.9
9	93.1	90.4	95.4	91.1	93.5	93.4	96.0	90.6
10	94.4	94.4	97.7	92.0	94.9	94.2	96.0	91.4
11	95.3	94.4	98.3	93.3	95.8	94.7	97.0	93.5
12	96.0	94.4	98.3	93.8	96.3	95.9	98.0	95.0
13	97.0	96.0	98.9	95.1	97.7	96.7	98.0	97.1
14	97.6	96.0	98.9	95.1	98.6	98.4	98.5	97.1
15	98.0	97.6	98.9	95.1	98.6	98.8	99.0	98.6
16	98.6	98.4	99.4	96.4	99.1	99.2	99.5	98.6
17	98.9	98.4	99.4	97.3	99.5	99.2	> 99.9	98.6
19	99.2	98.4	> 99.9	97.8	99.5	99.2	> 99.9	> 99.9
20	99.5	> 99.9	> 99.9	97.8	99.5	99.6	> 99.9	> 99.9
21	99.6	> 99.9	> 99.9	98.7	99.5	99.6	> 99.9	> 99.9
22	99.8	> 99.9	> 99.9	99.1	> 99.9	99.6	> 99.9	> 99.9
23	99.8	> 99.9	> 99.9	99.6	> 99.9	99.6	> 99.9	> 99.9

Table C9 continued

PHQ-9	Total	age 16-24	age 25-34	age 35-44	age 45-54	age 55-64	age 65-74	age 75+
26	99.9	> 99.9	> 99.9	99.6	> 99.9	> 99.9	> 99.9	> 99.9
27	> 99.9	> 99.9	> 99.9	> 99.9	> 99.9	> 99.9	> 99.9	> 99.9

SECTION D SENSITIVITY ANALYSES IMPUTATION

The following pages include a sensitivity analysis of some major analyses repeated on unimputed data.

Table D1

Means (M), standard deviation (SD), and group differences for the PHQ-9 items (unimputed data)

	Total		Male		Female		Group differences			
	M	SD	M	SD	M	SD	d [95% CI]	t	df	*p*
PHQ-9	2.68	3.87	2.37	3.68	2.95	3.99	-0.15 [-0.23,-0.07]	-3.66	2513	< 0.001
Little interest or pleasure	0.51	0.65	0.48	0.64	0.54	0.66	-0.09 [-0.17,-0.01]	-2.55	2513	0.011
Down/ depressed/ hopeless	0.38	0.63	0.33	0.59	0.42	0.65	-0.14 [-0.22,-0.07]	-3.61	2513	< 0.001
Sleep problems	0.41	0.68	0.35	0.62	0.47	0.72	-0.18 [-0.26,-0.1]	-4.58	2513	< 0.001
Tired/ little energy	0.47	0.70	0.39	0.65	0.53	0.73	-0.2 [-0.28,-0.12]	-5.11	2513	< 0.001
Poor appetite/ overeating	0.26	0.58	0.23	0.55	0.29	0.60	-0.1 [-0.18,-0.03]	-2.75	2513	0.006
Feeling bad about self/failure	0.19	0.52	0.18	0.51	0.19	0.53	-0.02 [-0.1,0.06]	-0.33	2513	0.744
Trouble concentrating	0.27	0.58	0.24	0.55	0.29	0.59	-0.09 [-0.17,-0.01]	-2.06	2513	0.039
Moving/speaking slowly - Fidgety/ restless	0.13	0.42	0.12	0.42	0.13	0.42	-0.02 [-0.1,0.05]	-1.12	2513	0.264
Suicidal ideation/ self-injury	0.07	0.30	0.07	0.30	0.07	0.30	0 [-0.08,0.08]	-0.33	2513	0.742

Table D2

Scale correlations: PHQ-9, GAD-7, BSI-18 (unimputed data)

Variable	M	SD	1	2	3	4	5
1. PHQ-9	2.65	3.84					
2. GAD-7	2.13	3.22	.85** [.84, .86]				
3. BSI GSI	4.25	7.52	.80** [.78, .81]	.76** [.74, .78]			
4. BSI Somatization	1.17	2.50	.65** [.62, .67]	.58** [.55, .61]	.87** [.86, .88]		
5. BSI Anxiety	1.22	2.50	.71** [.68, .73]	.72** [.70, .74]	.91** [.90, .92]	.73** [.71, .74]	
6. BSI Depression	1.86	3.36	.77** [.76, .79]	.73** [.71, .75]	.92** [.91, .92]	.65** [.63, .68]	.75** [.73, .77]

Note. PHQ-9 = Patient Health Questionnaire; GAD-7 = Generalized Anxiety scale; BSI GSI = Brief Symptom Inventory Global Severity Index; BSI Somatization = Brief Symptom Inventory Somatization Subscale; BSI Anxiety = Brief Symptom Inventory Anxiety Subscale; BSI Depression = Brief Symptom Inventory Depression Subscale; * indicates p < .05., ** indicates p < .001.

Table D3

Population based norms of the PHQ-9 (total sample – unimputed data)

PHQ-9	Total	age 16-24	age 25-34	age 35-44	age 45-54	age 55-64	age 65-74	age 75+
0	41.1	41.6	49.9	45.4	39.0	39.8	38.8	30.2
1	52.3	50.4	61.1	61.1	52.4	49.6	46.7	40.9
2	64.4	60.2	72.7	73.9	63.0	61.0	60.7	55.6
3	73.0	69.9	77.7	80.4	72.5	70.0	72.0	65.5
4	79.3	74.8	83.7	84.1	78.9	76.2	79.9	75.0
5	83.8	81.4	86.8	85.4	84.1	81.5	85.0	81.9
6	87.6	86.3	90.4	88.8	88.8	84.8	87.3	86.2
7	89.6	88.5	91.8	90.3	90.1	88.3	89.2	88.4
8	92.1	91.6	93.5	91.6	92.8	91.2	93.4	90.1
9	93.9	93.4	95.5	93.7	93.5	93.8	95.8	90.5
10	95.0	95.6	97.7	94.3	94.8	94.4	96.0	91.4
11	95.9	95.6	98.6	95.3	95.3	95.4	97.1	93.1
12	96.4	95.6	99.2	95.3	95.8	96.0	97.6	94.4
13	97.2	96.5	99.4	96.1	97.8	96.5	97.6	96.1
14	97.7	96.9	99.4	96.3	98.5	97.3	98.4	96.6
15	98.2	97.8	99.4	96.6	98.8	97.9	98.9	97.8
16	98.8	98.2	99.7	97.7	99.0	98.8	99.5	98.3
17	98.9	98.2	99.7	98.2	99.3	98.8	99.7	98.3
18	99.0	98.2	99.7	98.2	99.3	98.8	99.7	98.7
19	99.3	98.2	> 99.9	98.4	99.5	99.0	99.7	> 99.9
20	99.5	> 99.9	> 99.9	98.4	99.5	99.2	99.7	> 99.9
21	99.6	> 99.9	> 99.9	99.2	99.8	99.2	99.7	> 99.9
22	99.7	> 99.9	> 99.9	99.5	> 99.9	99.2	99.7	> 99.9
23	99.8	> 99.9	> 99.9	99.7	> 99.9	99.2	99.7	> 99.9
25	99.8	> 99.9	> 99.9	99.7	> 99.9	99.4	> 99.9	> 99.9
26	99.9	> 99.9	> 99.9	99.7	> 99.9	99.6	> 99.9	> 99.9
27	> 99.9	> 99.9	> 99.9	> 99.9	> 99.9	> 99.9	> 99.9	> 99.9

SECTION E SENSITIVITY ANALYSIS MEASUREMENT INVARIANCE

Table E1

Results of measurement invariance analyses (MLR estimator)

	χ^2	df	CFI	ΔCFI	RMSEA	ΔRMSEA	Measurement invariance
Gender (male, female)							
Configural invariance	1155.511	54	0.907	-	0.127	-	-
Metric invariance [1]	1206.264	62	0.903	-0.004	0.121	-0.006	✓
Scalar invariance [2]	1251.286	70	0.900	-0.003	0.116	-0.005	✓
Full invariance [3]	1319.321	79	0.895	-0.005	0.112	-0.004	✓
Age (<= 51, >51)							
Configural invariance	1175.743	54	0.906	-	0.128	-	-
Metric invariance [1]	1229.245	62	0.902	-0.004	0.122	-0.006	✓
Scalar invariance [2]	1275.067	70	0.899	-0.003	0.117	-0.005	✓
Full invariance [3]	1338.553	79	0.894	-0.005	0.113	-0.004	✓
Age * Gender							
Configural invariance	1247.458	108	0.903	-	0.13	-	-
Metric invariance [1]	1434.279	132	0.889	-0.014	0.125	-0.004	X
Scalar invariance [2]	1543.873	156	0.882	-0.007	0.119	-0.006	✓
Full invariance [3]	1833.261	183	0.859	-0.022	0.12	0.001	X
Income (<= 1750, >1750)							

Table E1 continued

	χ^2	df	CFI	ΔCFI	RMSEA	ΔRMSEA	Measurement invariance
Configural invariance	1183.111	54	0.905	-	0.129	-	-
Metric invariance [1]	1205.258	62	0.904	-0.001	0.121	-0.008	✓
Scalar invariance [2]	1234.491	70	0.902	-0.002	0.115	-0.006	✓
Full invariance [3]	1412.252	79	0.887	-0.014	0.116	0.001	X
Education (no university entry qual- ification, university entry qualifica- tion)							
Configural invariance	1189.642	54	0.904	-	0.129	-	-
Metric invariance [1]	1223.352	62	0.902	-0.002	0.122	-0.007	✓
Scalar invariance [2]	1231.751	70	0.902	0	0.115	-0.007	✓
Full invariance [3]	1256.479	79	0.901	-0.001	0.109	-0.006	✓

Note. All fit statistics are robust; CFI = Comparative Fit Index; CFI = CFI-differences for the different measurement invariance levels; RMSEA = Root Mean Square Error of Approximation; RMSEA = RMSEA- differences for the different measurement invariance levels; ✓= ΔCFI < -.010 complemented by RMSEA ≥ .015 indicates a violation of measurement invariance; marks measurement invariance for the respective level; [1] equivalency of thresholds; [2] equivalency of thresholds + factor loadings; [3] equivalency of thresholds + factor loadings + equivalency of constants; [4] equivalency of thresholds + factor loadings + equivalency of constants + unique-factor variances.

References

Kliem, S., Mößle, T., Rehbein, F., Hellmann, D. F., Zenger, M., & Brähler, E. (2015). A brief form of the Perceived Social Support Questionnaire (F-SozU) was developed, validated, and standardized. *Journal of Clinical Epidemiology*, *68*(5), 551–562. https://doi.org/10.1016/j.jclinepi.2014.11.003

Kocalevent, R.-D., Finck, C., Jimenez-Leal, W., Sautier, L., & Hinz, A. (2014). Standardization of the Colombian version of the PHQ-4 in the general population. *BMC Psychiatry*, *14*(1), 205. https://doi.org/10.1186/1471-244X-14-205

Shin, C., Ko, Y.-H., An, H., Yoon, H.-K., & Han, C. (2020). Normative data and psychometric properties of the Patient Health Questionnaire-9 in a nationally representative Korean population. *BMC Psychiatry*, *20*(1), 194. https://doi.org/10.1186/s12888-020-02613-0
